# An investigation of the effects of stage of ensilage on *Nassella neesiana* seeds, for reducing seed viability and injury to livestock

**DOI:** 10.1038/srep22345

**Published:** 2016-03-01

**Authors:** S. L. Weller, S. K. Florentine, J. F. Sillitoe, C. J. Grech, D. A. McLaren

**Affiliations:** 1 Centre for Environmental Management, Faculty of Science and Technology, Federation University Australia, Mount Helen, Victoria, Australia; 2 Research Services, Federation University, Mount Helen, Victoria, Australia; 3 DEDJTR Biosciences Research Division & School of Applied Systems Biology, La Trobe University, Bundoora, Victoria, Australia

## Abstract

The noxious weed *Nassella neesiana* is established on a wide range of productive land throughout southeastern Australia. *N. neesiana* seeds, when mature, are sharp, causing injury to livestock, thus posing a problem in fodder bales. To reduce infestations of agricultural weeds *in situ*, production of silage from weed-infested pastures is practised as part of integrated weed management (IWM). However, there is little data to demonstrate whether this process is useful to reduce infestations or the harmful properties of *N. neesiana*. Therefore, the minimum duration of ensilage required to reduce the viability of *N. neesiana* seeds was investigated, both with and without addition of ensilage inoculants in this process. Also, the decreasing propensity of the seeds to injure livestock, after various times and conditions of ensilage, was assessed. Ensilage inoculant reduced seed germination probability to zero after 35 days. When no inoculant was added, zero viability was achieved after 42 days. A qualitative assessment of the hardness of ensilaged seeds found seed husks were softer (and therefore safer) after 42 days, whether inoculant was used or not. Therefore, we suggest that both the viability of *N. neesiana* seeds and hardness of seed casings are significantly reduced after 42 days, thereby reducing the risks of seed dispersal and injury to livestock.

*Nassella neesiana* (commonly known as Chilean needle grass) is a noxious weed found in productive pasture areas of Australia that are increasingly being used as sources of emergency fodder. Emergency fodder is periodically required to fulfil the short-term needs of livestock producers who have been affected by drought, fire, or flood. The seeds of *N. neesiana* mature during the summer months and it is known that their local dispersal can occur because of adhesion to hay-making machinery and roadside mowing machines^1^,^2^. In addition, after being baled from an infested pasture, seeds can be widely dispersed in hay bales used for emergency fodder purposes, since quality control procedures are difficult to institute and bales can be consequently transported many miles into hitherto uninfested areas. Efforts to detect the presence of this species prior to hay making are thwarted by its ability to blend into the background matrix, resulting from its close resemblance to other pasture grasses^3^. This is of concern because the need for emergency fodder is an increasing feature of the livestock industry, and efforts are currently being made to utilise uncontrolled pastures without resorting to the use of economically and ecologically unviable herbicides. This investigation will examine the potential of duration of ensilage to (i) minimise soil seed bank input at the point of use of the baled material^4^,^5^, and (ii) to determine whether seed hardness or sharpness is affected through the process of ensilage, such that the physical danger to livestock of remnant material from the needle sharp seed casings is reduced or eliminated.

*N. neesiana* produces unpalatable panicle stems and seeds during late spring and early summer^6^ and the seeds have a sharp callus at the tip, which can cause significant injury to livestock since they are able to penetrate the skin or eyes, causing abscesses or blindness. The seeds also possess an awn, which becomes readily entangled in wool or hair, allowing the seeds to be dispersed on livestock as they move or are transported; for example, when the animals are shorn or removed from the pasture for slaughter^7^,^8^. While pastures infested with this weed can be safely grazed during the winter months when it is not in seed^9^,^10^,^11^,^12^, they are unsuitable for making pasture hay in the summer. The seeds can become dispersed within bales, or on machinery and personnel, because of the haymaking process. While the latter problem of dispersal on machinery and personnel is a local housekeeping issue, inclusions in hay bales is a significant dispersal vector, which requires strict control. In this respect, ensilage of mixed pasture grasses has been recommended as a method of producing a preserved fodder that is useful for addressing weed infestations without the need for herbicides. There is a small body of literature that demonstrates the effect of the ensilage process for preventing weed seed germination^5^,^13^,^14^,^15^,^16^, however little work has been done on *N. neesiana*.

Additionally, the mechanisms by which seed viability is extinguished through the ensilage process are of interest. Several researchers have investigated the use of commercial ensilage inoculants, which are substances that contain bacteria known to promote efficient conversion of harvested forage to silage, and which produce a variety of organic acids^17^,^18^,^19^,^20^,^21^. Some of these compounds, specifically lactic and acetic acids, have been implicated in seed viability extinguishment^14^, but have not been recently investigated and nor have they been investigated for their effect on *N. neesiana* seed viability.

Therefore, our objectives of this study were to: (i) determine the minimum time required to reduce the viability of seeds of *N. neesiana;* (ii) assess the viability of seeds of *N. neesiana* during and following ensilage, both with and without the addition of an ensilage inoculant; and (iii) qualitatively assess the effect of ensilage on the hardness of the callus (curved terminal spike) of *N. neesiana* seeds to determine if the this process renders them less likely to cause injury to livestock.

To determine the minimum time of ensilage required to achieve zero seed viability, as indicated by Viability Adjusted Germination (VAG) (defined in the “Methods” section, below), we used mini-silos^21^ to ensile mature *N. neesiana* seeds within a matrix of shredded and wilted pasture grass, for four different time periods. A commercial ensilage inoculant (a bacterial inoculum) was added to half of the samples, and seeds that had not been ensilaged were germinated as a ‘control’ to compare the effects of time of ensilage and addition of inoculum. To investigate the possible role of the metabolic products produced by both indigenous micro-organisms^22^ (i.e., those which are present on the plants, prior to harvest) and those from the bacterial inoculum in extinguishment of seed viability, we compared the production of relative amounts of three organic acids and ethanol, using High Pressure Liquid Chromatography (HPLC). To indicate whether the ensilage process reduced the hardness and sharpness of *N. neesiana* seeds, we qualitatively assessed the propensity of the sharp seed tip to bend or break off, in the seeds that had achieved zero viability. A chi-square test of contingencies was performed to determine expected frequency of germination, relative amount of bacterial metabolic products (organic acids and ethanol), and seed hardness after each stage of ensilage.

## Results

**Figure 1:**
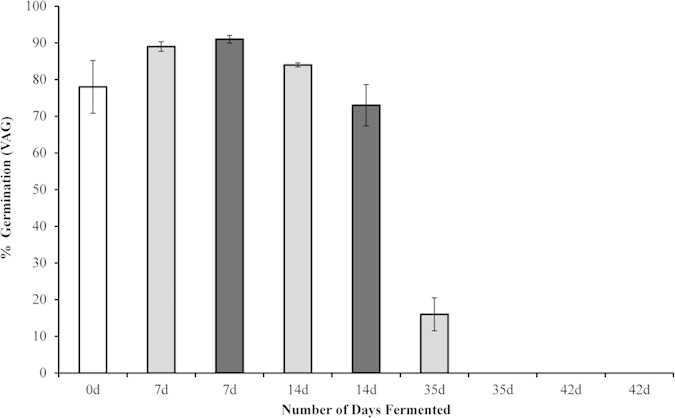
Viability adjusted germination percentages un-ensilaged seeds (control) and seeds ensilaged for varying times (days = d) at 20 °C, two inoculum treatments (added and not added). White bar = seeds not ensilaged (control), grey bars = no inoculum, black bars = inoculum added.

Figure 1 shows that whilst the duration of ensilage for short time periods (14 days or less) initially increased seed viability, compared to seeds that were not ensiled, viability was ultimately extinguished more rapidly in samples to which the inoculum was added. Extinguishment of seed viability, as indicated by VAG being reduced to 0%, occurred in 35 days when the ensilage inoculant was used, but not until 42 days when inoculant was absent.

Seven-day ensiled seeds showed the largest percentage viability, being 91% when inoculum was added and 89% when it was not. However, by 14 days, viability was reduced to 73% (inoculum added) compared to 84% (no inoculum). After 35 days, when the VAG of inoculated material was 0%, when inoculum was not used the VAG was reduced to 16% and the viability of un-ensiled seeds was 78%.

A chi-square test of contingencies for the expected frequency of germination indicated that the use of inoculum significantly reduced the time required to achieve extinguishment of seed viability; χ^2^ (α = 0.05, 3 d.f., N = 431) = 74.429, p < 0.001. Additionally, ϕ (phi), which is a measure of the association between change of germination frequency with time and inoculum use, was moderately strong, ϕ = 0.416. Therefore, we suggest that the addition of inoculum significantly reduces the time required for seeds to become unviable.

**Figure 2:**
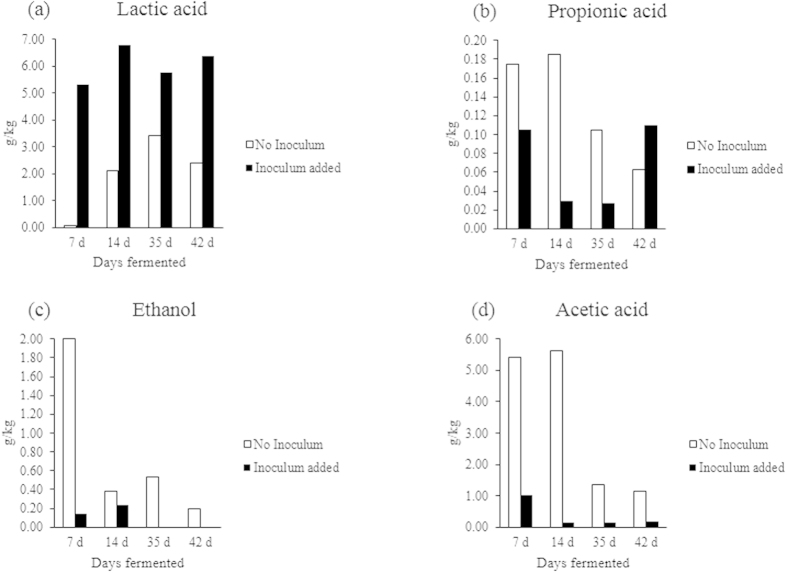
(**a–d**) Results of HPLC analysis for concentrations of metabolic products of ensilage in samples fermented for 7, 14, 35 and 42 days; (**a**) Lactic acid, (**b**) Propionic acid, (**c**) Ethanol and (**d**) Acetic acid. White bars indicate ‘No inoculum’ treatment; black bars indicate ‘inoculum added’ treatment.

Completeness of the ensilage process and the effectiveness of the addition of ensilage inoculant, as indicated by the increase in concentrations of lactic acid, acetic acid, propionic acid, and ethanol, demonstrated that the addition of inoculum produced higher concentrations of lactic acid compared to other organic acids. Butyric acid, which is an indicator of adverse ensilage outcomes, was also tested for but not found in any of the samples. Figure 2(a,d), compares the concentrations of lacticacid, acetic acid, propionic acid and ethanol found in the inoculated and uninoculated ensilage.

In samples to which inoculum was added, consistently high concentrations of lactic acid, a desirable bacterial metabolic product for silage preservation^4^,^14^, were present across the entire ensilage period; by contrast, in samples to which no inoculum was added, considerably lower concentrations of lactic acid were present (Fig. 2a). The relative concentrations of the three other metabolic products were less consistent between inoculum treatments over the entire ensilage time period (Fig. 2b–d).

A chi-square test of contingencies indicated that use of an inoculum significantly influenced the production of lactic acid; χ^2^ (α = 0.05, 3 d.f., N = 32154) = 2444.209, p < 0.001. However, the association between amount of compound produced and inoculum use was moderately low ϕ = 0.276. Notwithstanding this, sufficient quantity of lactic acid was produced to ensure efficient silage preservation, so the use of commercial inoculum is positively indicated. By contrast, not using an inoculum resulted in relatively higher concentrations of acetic acid, propionic acid and ethanol^4^,^21^,^22^,^23^,^24^.

**Figure 3:**
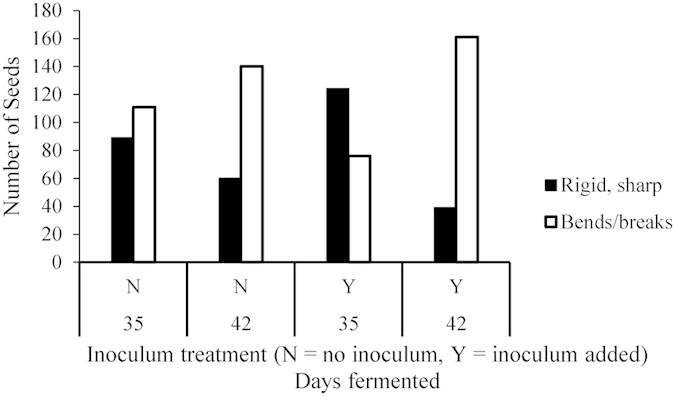
Qualitative change in seed sharpness after 35 and 42 days of ensilage. Black bars indicate numbers of seeds with sharp, rigid seed tip that penetrated the skin of experimenter’s finger. White bars indicate numbers of seeds which tip of seed bent or broke off completely upon contact with skin.

Figure 3 shows that after 35 days of the ensilage process, the use of inoculum did not appear to significantly reduce seed rigidity. This contrasted with seeds fermented for 42 days, in which hardness or sharpness of the seed callus was reduced, whether inoculum was used or not, since there was a marked tendency for seed callus to either bend or break off completely. These results do not follow a strong trend, however.

A chi-square test of contingencies, to determine whether seed hardness or sharpness decreased with the use of an inoculum, indicated a significant difference between ensilage period and inoculum use, χ^2^ (α = 0.05, 3 d.f., N = 800) = 85.792, p < 0.001. Although the association between a reduction in seed hardness/sharpness and inoculum use was moderately low, ϕ = 0.327, given that more seeds in total were softer by 42 days than 35 days, the combination of addition of inoculum and time of ensilage does contribute to the reduction in hardness and sharpness of these seeds.

## Discussion

A reduction in viability of *Nassella neesiana* seeds correlated strongly with length of time of ensilage. However, complete extinguishment of viability was not observed until after either 35 days (five weeks) (inoculum added) or 42 days (six weeks) (no inoculum) of ensliage, whereas the minimum time period in which a useable silage may be produced, under ideal conditions, can be less than either of these time periods. Research investigating only the production of silage as fodder, without consideration of any weed issues, indicates that under ideal conditions ensilage processes which result in a nutritious end product can be complete in as little as three to four weeks (21 to 28 days)^4^. These findings underscore the importance of allowing the ensilage process to run its course, rather than to use partially ensilaged material for emergency relief fodder.

By contrast, previous investigations into the reduction or extinguishment of viability by ensilage, for the seeds of other weed species, have shown that this takes place following longer time periods, and can be up to eight or twelve weeks (56 or 84 days)^5^,^13^. These investigations found that species of grass seeds, in particular, were unviable after these periods of ensilage, which supports the findings of the current research into *N. neesiana*.

From these results, it appears that the shortest expected time period of ensilage to produce fodder for livestock, as little as three to four weeks (21 to 28 days), is much less than the time required to extinguish *N. neesiana* seed viability. This finding indicates that silage made from pastures infested with this weed should perhaps be quarantined for consumption for a minium of six to eight weeks (42–56 days) to ensure that seed viability is extinguished, whether inoculum is used or not.

The application of ensilage inoculant to reduce the viabiltiy of the seeds of *N. neesiana* in silage is also positively indicated, with the minimum necessary time period to produce silage that is unlikely to contain viable seeds of this weed being shortened by the use of such inoculants. Weed seed germination was reduced to effectively zero (VAG = 0%) by five weeks, rather than six, when the ensilage inoculant was used. There is also a positive indication of the development of favourable ensilage profile in the grass species used in this experiment, since relatively more lactic acid was produced with the use of ensilage inoculant. Other researchers have concluded that the production of higher concentrations of lactic acid relative to other organic acids ensures longevity of silage preservation^17^,^18^,^19^,^20^,^21^,^24^.

To the present time, however, there has been little research into the role of lactic acid in silage, other than as a indicator of positive outcomes of fodder preservation. For example, whether this compound is the mechanism responsible for reducing or eliminating weed seed viability, or whether it is a simple by-product of fermentation, has been not been rigourously examined. The only apparent contribution to this is a study early in the 20^th^ century by Tiledesley (1937) on the effect on seed viability of ‘silage juice’^14^. It was commented in this paper that non-volatile lactic acid was a possible agent responsible for extinguishing seed viability^14^. Since the concentrations of lactic acid were higher in samples to which inoculum was added, and that the time period for seed viability extinguishment was shortened when an inoculum was used, it is possbile that this compound is, at least in part, responsible for this accellerated seed death.

Another possible explanation of seed death during ensilage, is the presence of carbon dioxide in the samples. Tildesley (1937) noted that seeds placed at the top of a silo were still viable following a period of ensilage, in contrast to seeds ensilaged in the middle and at the bottom of the silo^14^. In this study, Tildesley found lower concentrations of carbon dioxide correlated with lower concentrations of organic acids near the silage/air interface at the top of the silo, and proposed this observation as contributing to the explanatory mechanisms for this phenomenon^14^. Because the appropriate equipment was unavailable, the current research did not investigate whether this mechanism was responsible for seed death of *N. neesiana* seeds, but this could be a useful investigation for other researchers.

From the foregoing, we suggest that the use of an inoculum for both forage preservation and reduction in seed viability is positively indicated, since there are multiple benefits. These include: shortened time of forage preservation due to the guaranteed presence of lactic acid producing bacteria in the system; longevity of storage due to the presence of higher concentrations of lactic acid, rather than acetic acid; lower numbers of spoilage organisms, such as competing species of bacteria and yeasts; and a more rapid reduction of weed seed viability that occurs when inoculum is not used.

The sharpness of the seeds of *N. neesiana* is of significant concern to livestock producers, since these can cause debilitating injuries to livestock^25^. The results from the present study appear to indicate that ensilage of the seeds of *N. nee*siana results in an overall reduction of seed sharpness, with the time for which the processes of ensilage are allowed to occur being, perhaps, the most significant factor. There did not, however, appear to be a strong indication of whether or not the addition of ensilage inoculant has any effect on the reduction of time needed for this process.

Although a reduction in seed hardness and sharpness because of ensilage is positively indicated, these results are not completely conclusive. Therefore, this area may be of future interest for other researchers. It is recommended that a suitable testing regimen or equipment be developed to test seeds of this weed and others of a similar morphology for their propensity to injure livestock. This would make it possible to test the effect of ensilage upon seed hardness and/or sharpness more precisely. In addition, the investigation of whether longer times of ensilage contribute to the reduction of seed hardness and/or sharpness is required.

In conclusion, the results obtained from the present study indicate that whilst ensilage is able to prevent germination of seeds of *N. neesiana*, this requires a minimum time of at least five weeks of ensilage, with the use of an inoculum. It would, therefore, be unwise to rely on an assumption that short periods of ensilage, for example two to three weeks, will achieve both a ‘good quality’ fodder product and that seeds of this weed will not be viable and not injure livestock. We recommend that ensilage should be allowed to occur for a minimum period of three months in order to achieve all three outcomes of silage preservation^4^, extinguishment of weed seed viability^5^,^13^,^16^, and reduction or elimination of seed casing hardness to prevent injury to livestock.

## Methods

Mature seeds of *N. neesiana,* were harvested from approximately 250 plants during November 2010 from Broadmeadows Valley Park, Melbourne, Victoria. Seeds that were judged to be ‘filled’ were placed in labelled brown paper bags and stored at room temperature until use. Filled seeds were visually intact, and were firm to gentle squeezing, suggesting that the starch grain was firm and full. In this way seeds that had an inadequate starch store, which is required for germination and early growth, were eliminated.

The seeds were cut from the awns with small dissecting scissors, before being counted into lots of 25 and placed into small bags of nylon tulle with a mesh size of 0.5 mm, approximately half the diameter of the seeds. This allowed the seeds to be fully exposed to the ensilage processes within the grass material without becoming lost from the nylon bags.

The grass material used for ensilage was a mixture of leaves and stems of *Dactylis glomerata* (Cocksfoot) and *Phalaris aquatica* (Phalaris), sourced from a property in Oaklands Junction, Victoria, Australia. The plants were harvested at their early seeding stage so that the concentrations of stored starches were near to their maximum values, in order to optimise ensilage^24^. Approximately 16 kg of pasture grass was collected in total by hand harvesting. The stems were cut with hand shears to approximately 10 cm above ground level to ensure no soil would be included, since the presence of soil in silage increases the risk of inclusion of *Clostridia* bacterial species, which are known to interfere negatively with the production of lactic acid and thus result in spoilage^4^,^24^. Harvested grass material was transported to a laboratory environment within two hours, where it was shredded. Following standard silage procedure, the shredded material was allowed to wilt for 48 hours at room temperature^4^ before being divided into two approximately 8 kg equal portions. The first portion was kept as a control sample. The second was treated with one litre of an aqueous suspension of a commercial ensilage inoculant. The inoculant, Pioneer^®^ 1127 Grass/Legume Silage Inoculant (1.25 × 10^11^ CFU/g), was supplied by Pioneer Hi-Bred Australia Pty. Ltd. This inoculant contains the microbes *Lactobacillus plantarum* and *Enterococcus faecium* in a dried form and was sprayed with a garden sprayer (dilution rate of 0.008 g of inoculant per litre of tap water) onto the material in three applications. The rate of application of inoculant per kilogram of grass was 1.3 × 10^8^ CFU/kg. Between each application, the material was thoroughly mixed in order to ensure even distribution of the inoculant and to prevent local wet spots. The two samples were then divided into sixty four 250 g portions, which were placed into food-grade (nylon/polythene) vacuum sealable bags. Into each 250 g portion, one mesh bag containing approximately 25 *N. neesiana* seeds was placed in the centre and the bags were vacuum sealed. Four ensilage times were designed, these being 7, 14, 35 and 42 days.

The experimental setup of all ensilage treatments is given in Table 1. At the end of each ensilage period, the eight bags of each ‘day’ treatment were opened and the mesh bags containing *Nassella neesiana* seeds were removed and made ready for germination trials. The mesh bags of seeds were placed in small plastic zip-lock bags containing approximately 50 ml of distilled water and allowed to soak for one hour to become saturated. The seeds were then removed from the mesh bags and placed in Petri dishes lined with filter paper and moistened with distilled water. Four Petri dishes of each day treatment were placed into two large zip-lock bags (43 × 30 cm) and placed into one of two temperature-controlled environmental chambers at 20 °C. The first chamber was set with a 12 hours light/12 hours dark cycle, whilst the other had 24 hours continuous darkness. To assess germination, the seeds were inspected weekly, and any germinated seeds were counted and removed. For this investigation, germination was considered to have occurred when the radicle had emerged from the seed by between 2–5 mm. Seeds that did not germinate after 49 days were assessed for viability with tetrazolium test^26^, and the numbers of germinated and viable seeds were then used to calculate Viability Adjusted Germination (VAG), which is the total percentage of viable seeds that germinated, using the following formula:


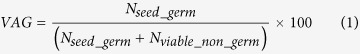


Where: N_seed_germ_ = number of seeds germinated, and, N_viable_non_germ_ = number of seeds which did not germinate, but were viable.

To determine how completely the grasses in the bulk of the silage had fermented, and whether an appropriate outcome was obtained, analysis of the concentrations of five ensilage metabolic products, lactic acid, acetic acid, propionic acid, butyric acid, and ethanol, was conducted using high performance liquid chromatography (HPLC)^27^. This analysis used a HiPlex-H (organic acid) column, set at a temperature of 80 °C as the stationary phase, and a solution of 0.005 M H_2_SO_4_ (sulfuric acid) at a flow rate of 0.7 mL per minute as the mobile phase. An ultraviolet detector, set for a wavelength of 236 nm, was used for the measurement of acids and ethanol as they emerged from the column, and analytical grade standards of lactic acid (1.5 g/kg), propionic acid, butyric acid, and ethanol (0.5 g/), and acetic acid (10.0 g/kg) were available for comparison. Analyses of all standards and samples were run for 60 minutes each.

After each of the designated ensilage periods (7, 14, 35 and 42 days) and following removal of seeds, one replicate bag of the ensiled grass samples was squeezed to remove as much air as possible, packed into a labelled zip-lock bag and frozen within five minutes of the sample being opened to ensure no further changes would occur before analysis. Prior to testing for organic acids and ethanol, the samples were removed from the freezer for two hours allowing them to thaw at room temperature. A sub-sample of 25 mL of ensiled material was taken, measured by packing this amount firmly into a small beaker. The ensiled material was transferred to a 250 mL (capacity) ‘Schott’ brand glass bottle with a liquid-tight plastic screw cap, to which 250 mL of MilliQ water was added. The bottles were placed on a shaking table for one hour, removed, and allowed to settle at room temperature for one hour. Approximately 40 mL of the supernatant liquor was drawn off with a syringe and a few millilitres of each sample was filtered through a Phenomenex® syringe filter (0.47 μm pore size) into labelled sample vials, which were securely capped. These vials were placed into the auto sampler of the HPLC, together with standards of lactic, acetic, propionic, and butyric acids, together with ethanol, to give comparative ensilage concentrations.

Seeds fermented for 35 and 42 days were rated for sharpness in a qualitative manner by holding the seed with forceps and pressing the tip of the seed into the underside of the tip of the index finger of the experimenter. The propensity of the seed tip to penetrate the skin, bend on contact with skin or break off completely was recorded.

## Additional Information

**How to cite this article**: Weller, S. L. *et al.* An investigation of the effects of stage of ensilage on *Nassella neesiana* seeds, for reducing seed viability and injury to livestock. *Sci. Rep.* **6**, 22345; doi: 10.1038/srep22345 (2016).

## Figures and Tables

**Table 1:** Summary of numbers of treatments, numbers of replicates and samples in silage treatment of seeds of *Nassella neesiana*.

Ensilage time (‘Day’ treatment)	Ensilage inoculant added	Replicates	Total samples
7	No	8	64
14	No	8	
35	No	8	
42	No	8	
7	Yes	8	
14	Yes	8	
35	Yes	8	
42	Yes	8	
